# Social and environmental analysis of food waste abatement via the peer-to-peer sharing economy

**DOI:** 10.1038/s41467-020-14899-5

**Published:** 2020-03-10

**Authors:** Tamar Makov, Alon Shepon, Jonathan Krones, Clare Gupta, Marian Chertow

**Affiliations:** 1 Guilford Glazer Faculty of Business and Management, Ben-Gurion University of the Negev, Be’er Sheva, Israel 8410501; 2 Center for Industrial Ecology, School of Forestry & Environmental Studies, Yale University, New Haven, CT 06511 USA; 3 Department of Nutrition, Harvard T.H. Chan School of Public Health, Boston, MA 02115 USA; 4 Department of Earth and Environmental Sciences, Boston College, Chestnut Hill, MA 02467 USA; 5 Department of Human Ecology, University of California Davis, Davis, CA 95616 USA

**Keywords:** Environmental impact, Sustainability

## Abstract

Reducing food waste is widely recognized as critical for improving resource efficiency and meeting the nutritional demand of a growing human population. Here we explore whether the sharing economy can provide meaningful assistance to reducing food waste in a relatively low-impact and environmentally-sound way. Analyzing 170,000 postings on a popular peer-to-peer food-sharing app, we find that over 19 months, 90t of food waste with an equivalent retail value of £0.7 million were collected by secondary consumers and diverted from disposal. An environmental analysis focused on Greater London reveals that these exchanges were responsible for avoiding emission of 87–156t of CO_2_eq. Our results indicate that most exchanges were among users associated with lower income yet higher levels of education. These findings, together with the high collection rates (60% on average) suggest that the sharing economy may offer powerful means for improving resource efficiency and reducing food waste.

## Abstract (web summary)

Reducing food waste is important. Here the authors investigate whether digitally enabled social networks can efficiently reduce food waste and find that in 19 months, around 91t of food waste were collected by secondary consumers and diverted from disposal.

## Introduction

Global postharvest loss of edible food is estimated at 1.3 billion metric tons (t) annually^1^. Food loss limits society’s ability to sustainably feed a growing population and squanders resources on a grand scale^2–7^. Further, the production of food that is intended for human consumption but not eaten accounts for 8% of global anthropogenic greenhouse gas (GHG) emissions, 20% of fresh water consumption, and 30% of global agricultural land use^8,9^. Although food is lost across the entire supply chain, in high-income countries losses occur disproportionally at the post-retail and consumer levels^1,8,10^. Such losses, referred to hereafter as food waste, range between 124 and 154 kg per capita per year and come at a high economic cost, estimated at 10–25% of households’ annual food expenditures^11–15^. Waste at the retail level is generated in large volumes in part because of market, regulatory, and sociocultural standards for food quality, aesthetics, safety, and abundance (e.g., retailers desire to present fully stocked shelves)^16,17^. At the household level, food waste is the outcome of multiple behaviors, including inefficient household food management, confusing expiry date labels, and over-purchasing^18,19^. Altogether, the massive waste of resources and related environmental impacts have made food waste recovery an important environmental mitigation strategy and one of the sustainable development goals (12.3)^14,20,21^.

At the same time, food insecurity and malnutrition affect not only low- and medium-income countries, but high-income ones as well. Estimates suggest that 12.7% of US households were food insecure in 2015, meaning that their “access to adequate food was limited by a lack of money or other resources”^22^. Over 10% of UK residents experienced food insecurity in 2014 and the recent tripling of food bank activity has been taken by many as an indication that food insecurity is on the rise^23,24^. Since 70–86% of food waste at the retail and household levels is thought to be still edible when discarded, redistributing edible yet unwanted food from primary to secondary consumers has been put forward as a win–win solution for simultaneously tackling food waste and food insecurity^11,15^.

In recent years, the sharing economy—as implemented via decentralized, peer-to-peer (P2P) networks of mobile apps and users—has become increasingly popular. Owing to technological developments and the ubiquity of GPS-enabled smart devices, platforms such as Airbnb have created alternative markets by efficiently matching agents with excess asset stocks (e.g., cars and apartments) with potential consumers^25^. While some platforms facilitate sharing activities for a fee (e.g., Airbnb), other platforms (e.g., CouchSurfing) support free P2P exchanges of goods and services.

Although sharing underutilized assets (with or without pay) is a longstanding practice, the low transaction costs and barriers-to-entry of digital platforms have made sharing cheaper and easier than ever and therefore possible on a much larger scale. In particular, the scalability, flexibility, and potential for high-speed exchange make digital sharing platforms well-suited for the exchange of perishable food items^21^. Given that both surplus and unmet demand for edible food at the household level coexist in the same geographies, sharing platforms could help match unwanted food with secondary customers and deliver environmental and social benefits.

Despite growing interest in P2P food-sharing networks, their potential to reduce food waste in a sound and sustainable manner remains questionable. First, while sharing platforms in theory can help redistribute unwanted food efficiently, whether there is indeed enough interest in such exchanges from both providers and collectors requires empirical validation. In contrast to other secondhand products (e.g., vehicles) or even food surplus offered by farmers or distributors, food waste has low (or even negative) economic value. As a result, providers may not be willing to incur the transaction costs—the time and effort involved—associated with P2P exchanges. Similarly, supply constraints (in terms of both volume and variety of food) might lower demand for food waste, as disappointed collectors who cannot find a good match for their tastes or needs abandon the platform^21^. Psychological barriers might also limit demand for food waste: research shows that people often feel disgust toward non-new items^26^ and have misgivings about consuming recycled water and crops produced using recycled water^27^.

Second, even if the sharing economy is successful at facilitating P2P food exchanges, the broader environmental implications of such exchanges are not well understood. Although the sharing economy is generally assumed to reduce environmental burdens^28^, some question this premise. Recent work reveals that sharing economy platforms can actually stimulate new demand for durable assets, such as cars^29^ and housing^30^ as well as services, including tourism and road transport^31,32^. Similarly, food sharing might lead to an overall increase in food consumption. In addition, added transport related to food exchanges could potentially negate the environmental benefits associated with food waste reduction.

Finally, food sharing might not be a viable way to address food insecurity, as some have suggested^33,34^. Irrespective of any ethical considerations regarding the appropriateness of using food waste to feed those experiencing food insecurity^35^, research examining the social dimensions of the sharing economy indicates that higher income groups rather than lower ones often benefit more from participation^25^. While food insecure populations tend to have a lower average income and in some cases lower educational attainment^36–38^, participants in free sharing platforms tend to be highly educated; high cultural capital is potentially a prerequisite for successful participation^39^. Thus, it remains unclear whether digital platforms for food sharing are actually used by and benefit people who are experiencing food insecurity.

Analyzing data provided by OLIO, a popular P2P food-sharing platform (See Supplementary Note 1 for more), we examine the types, weights, and retail value of foods offered and shared, quantify the associated environmental impacts, and investigate the socioeconomic characteristics of the platform’s user network. Our results reveal that during the period examined, 90t of food waste (60% of all items offered) with an equivalent retail value of up to £750,000 were collected and diverted from disposal. Exchanges were predominantly among users associated with lower income yet higher levels of education. Finally, analysis of exchanges in Greater London suggests that the environmental benefits associated with avoided food waste outweigh the costs associated with added transport.

## Results

### Food exchanges typology

**Fig. 1:**
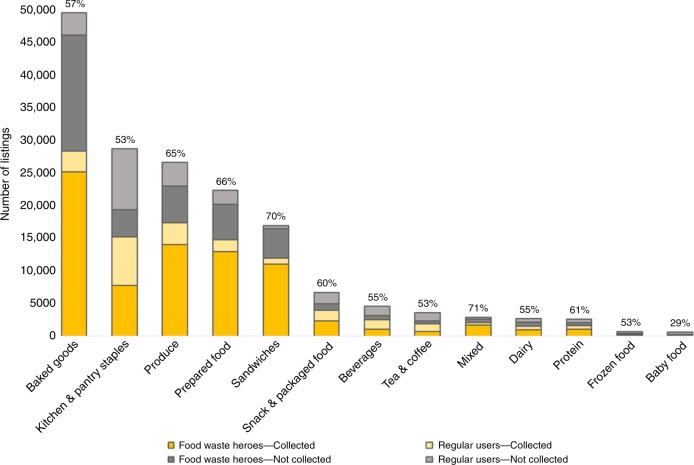
Listings and collections by food category and user group (food waste hero vs. regular user) of the full OLIO network. Percent shown on top of bars report overall collection rate in each category; yellow colors represent collected items, while gray colors represent uncollected items; darker shades (in both yellow and gray) represent items listed by food waste heroes, while lighter shades represent items listed by regular users.

Of the over 170,000 listings of food items offered on OLIO between April 2017 and October 2018, we find that 60% were successfully collected (see Fig. 1). Over this time period, 91 ± 1t of food (5th and 95th Monte Carlo (MC) percentiles, respectively) with an estimated retail value of £720–750 thousand were diverted from waste and passed on to secondary consumers across the entire OLIO network (see Supplementary Table 1a, b).

The types of foods listed and collection rates by food category are presented in Fig. 1. The most commonly listed items were baked goods (29%), kitchen and pantry staples (17%), fresh produce (16%), and prepared food (13%). Collection rates varied by category and were highest for mixed listings (71%), sandwiches (70%), prepared food (66%), and fresh produce (65%). All food categories had collection rates of 53% or higher, other than baby food, where only 29% of listings were collected (see Supplementary Table 2a, b, and Supplementary Note 2).

Network analysis investigating interactions among platform users reveals that of the 22,000 users who participated in at least one food exchange, 12% had both received and given at least one item, 26% had only given items, and 62% had only collected. Specifically, in each local network examined, a small number of mega-providers (represented by larger green nodes in Supplementary Fig. 1) provided most of the food changing hands. These mega-providers were often food waste heroes: official OLIO volunteers who collect surplus food from local businesses (e.g., supermarkets or delis) and post them for collection via the platform. Overall, heroes were the main sources of supply on the platform, responsible for 71% of all collected and non-collected listings. Heroes provided most of the supply of ready-to-eat foods (e.g., sandwiches and baked goods), while regular users were primarily engaged in sharing pantry and frozen food items. Furthermore, listings offered by heroes had better collection rates overall (66%) compared to listings posted by regular users (47%). This held true across all food categories including those more frequently supplied by regular users (see Supplementary Table 2b). Food waste heroes were also more likely to engage with a larger number of users (27 on average compared to 2.5 for regular users) and take part in reoccurring exchanges. To wit, 79% of the time, heroes provided food to a user they had interacted with previously (compared to 31% for regular users).

### Environmental impacts

**Fig. 2:**
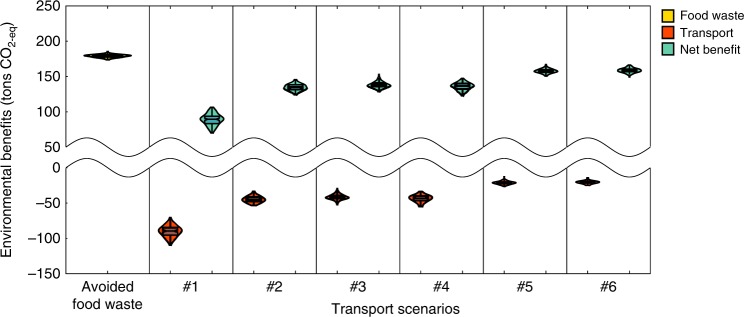
Environmental burdens and benefits by travel scenario (in GHG emissions). Leftmost section represents environmental benefits associated with avoided food waste in terms of avoided GHG emissions (yellow violin plot- constant across all scenarios). Each of the other six sections show environmental costs related to added travel (red violin plots), and net environmental benefits (green violinplots) associated with OLIO exchanges by travel scenario #1–6 (see Table 1), in terms of GHG emissions.

Figure 2 presents results for an environmental analysis of P2P sharing in the Greater London area. We compare the full life-cycle GHG benefits of avoided food waste with the emissions associated with added road transport related to exchanges according to six transport scenarios (see Table 1 and Supplementary Table 3a–d). Overall, 41 t of food waste (with a 5th and 95th percentiles interval of [40, 42 t]) were exchanged via the platform in Greater London during the study time period. This activity involved between 226 and 451 thousand added kilometers by car or London bus depending on travel scenario (see Table 1 and Methods). Under all travel scenarios considered, the reduction in GHG emissions due to avoided food waste was larger than the added GHG emissions related to additional road travel.

**Table 1:** Travel scenarios.

Scenario	Transport mode	Assumptions
#1	Car	Two-way dedicated trips
#2	Car	One-way dedicated trips
#3	Car	One-way dedicated trips, walk <1.6 km
#4	London bus	Two-way dedicated trips
#5	London bus	One-way dedicated trips
#6	London bus	One-way dedicated trips, walk <1.6 km

Specifically, food exchanges were associated with a net benefit in terms of GHG emissions ranging from a minimum of 87t of CO_2_eq ([72, 102 t CO_2_eq]) under Scenario #1 (a boundary condition where collectors were assumed to drive back and forth by car only to collect a single food item), to a maximum of 156 t of CO_2_eq ([150, 162 tCO_2_eq]) in Scenario #6 (the scenario closest to the average London transport mix). A net benefit was also observed across all scenarios investigated in the sensitivity analysis with the exception of the most carbon-intensive travel scenario (#1) (see Supplementary Table 4a–c and Supplementary Note 3). It is important to note that these benefits take into account only cradle-to-grave emissions associated with food waste. When considering indirect land use change or what has been defined as the “carbon opportunity cost” of avoided food production, environmental benefits are found to be roughly five times larger than our current estimates (see Supplementary Table 5)^40^.

Critically, we assumed that food acquired via the platform fully displaces purchases of identical “new” foods. For example, when a collector receives a loaf of bread from another peer, we assumed that she would then purchase one less loaf of bread that week. Since this might not always be the case, we examined the minimal displacement rate needed so that platform activity would generate a net benefit. Back-of-the-envelope calculations reveal that if 12–50% of the food exchanged displaces purchases of identical food items, OLIO would generate net environmental benefits, depending on the transport scenario (scenarios #6 and #1, respectively).

### Social analysis

**Fig. 3:**
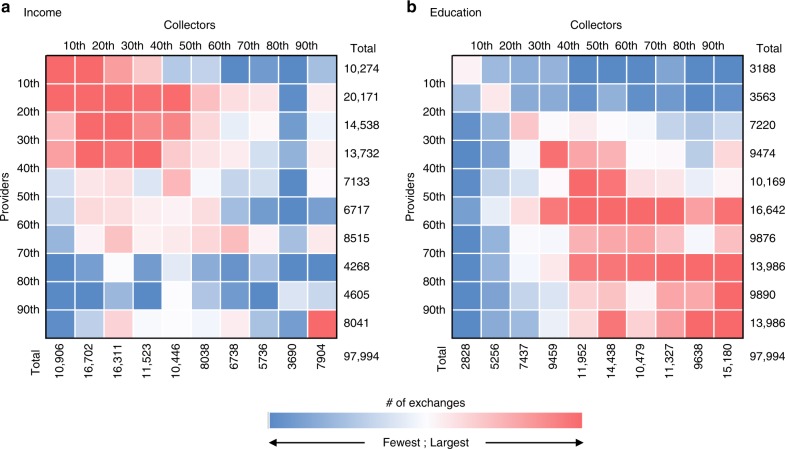
Socio-demographic analysis of food flow for all UK exchanges. Panel **a** represents exchange frequency by providers’ (vertical) and collectors’ (horizontal) income percentile. Panel **b** represents exchange frequency by providers’ (vertical) and collector’s (horizontal) education percentile. In both panels, exchange frequency ranges from the least amount of exchanges (dark blue) to the largest number of exchanges (dark red).

Figure 3 presents exchanges between providers and collectors according to the income and education levels representative of each user’s home address (see Methods). As is evident from the red clusters in each panel, most exchanges mediated by the platform were conducted between users from areas with relatively low income levels and high education levels.

## Discussion

Reducing food waste is widely recognized as critical for improving resource efficiency and meeting the nutritional demand of a growing human population. Finding ways to improve utilization of food waste could deliver environmental, social, and economic benefits. Here, we have analyzed data from a popular smartphone app and illustrate that the sharing economy could help divert food waste from disposal by efficiently matching unwanted food items with secondary consumers. The high collection rate of foods with a short shelf life (e.g., sandwiches, fresh produce, and prepared foods) suggests that P2P sharing could offer new reuse paths for foods that are typically deemed unsuitable for centralized food-redistribution channels in part because they are required to abide by various standards (e.g., following cold chain requirements during storage and transport)^41,42^.

While the high collection rates, topping 70% in some food categories, indicate that there is currently ample demand for food waste, the total amount of food waste diverted across the entire network remains, for now, marginal. As such, the real promise lies in scaling up P2P exchanges of food waste. Since various barriers on both the supply and demand side could limit people’s willingness to participate, more work is needed to ascertain not only what the practical limits are to food sharing but also how such expansion could occur.

Regarding environmental impacts, we show that the benefits of avoided food waste outweigh the burdens associated with added road transport. Consequently, food sharing offers a net GHG benefit that is equivalent, on average, to 0.6% of London users’ per-capita food-related emissions (assuming a 2.7 t CO_2_eq per cap annually associated with food consumption in London)^43^. We confirm the robustness of our key finding that the GHG benefits of food sharing outweigh the costs, by examining alternative emissions coefficients reported in the literature. In all but the worst-case transport scenario (#1), in which collectors are assumed to drive back and forth by car to make a collection, the benefits of food sharing outweigh the added climate burdens. Since there are only 0.3 cars per adult in the greater London area^44^, scenario #1 is not very realistic in this geography. A transport scenario based on the average London transport mix (21% walking, 34% car, and 45% public transport^45^) suggests that travel GHG burdens are likely closer to scenario #6 (20 tCO_2_eq with an interval of [16, 24 t CO_2_eq]). Moreover, if OLIO collection is done as part of routine trips (for commuting or shopping) or if more than 20% of collections are conducted on foot, the added emissions would be even lower. Nonetheless, scenario #1 represents an important boundary condition that could be highly represented in areas with limited public transport options or suburban and rural communities where travel distances tend to be longer. More work is needed to quantify the environmental implications of food sharing in non-urban areas.

Our environmental analysis has several limitations. First, the data does not contain information at the sub-listing level. Thus, when listings contain more than one food item (e.g., ten loaves of bread) we could not definitively ascertain whether all ten loaves were collected or only nine. Anecdotal information provided by platform managers suggested that roughly 90% of all multi-item listings are collected in full and thus we assumed that to be the case.  Second, since no historical data on user location was available (due to privacy concerns) we used notification location as a proxy for collectors’ points of origin. Hence, there is uncertainty involved in estimating travel routes and distances. Third, in our analysis we employ a general GHG emissions factor assuming that the types and quantities of foods shared via the platform are similar to the average UK food waste composition. However, since the environmental benefits would be greatest if the platform facilitated exchanges of carbon-intensive foods (e.g., meat-based products) a more granular examination focusing on the factors which facilitate successful exchanges of such foods could help maximize the environmental benefits delivered via food sharing. Finally, the results of our environmental analysis are contingent on the fact that food obtained via OLIO fully displaces purchases (and consequently production) of “new” food items. Since, however, non-new products seldom displace purchases of new products on a 1:1 basis, this might not be the case ^46,47^. Therefore, while food sharing might have social or economic benefits, it is important to consider the minimal displacement rate needed to ensure that the environmental benefits of food sharing remain higher than the environmental burdens incurred by this activity.

While low substitution rates might hinder the GHG benefits derived by OLIO, adding the carbon opportunity cost (COC) of indirect land use changes could increase the calculated GHG benefits considerably. As more data on the COC^40^ in the context of the UK become available, the uncertainties associated with the overall GHG emissions of food waste and of food sharing will be reduced.

Another aspect to consider is that if food sharing were to reduce consumers’ food purchases, the resulting economic savings might lead to rebound effects^48^. With an estimated retail value of £0.7 million, exchanges of food waste via OLIO could lead to meaningful respending effects, and increase, rather than decrease, overall GHG emissions ^49^. Rebound effects are particularly relevant for people in lower income brackets, such as those experiencing food insecurity, who tend to spend any additional income on fulfilling basic family needs. Since sharing economy platforms are free of the social stigma associated with seeking help from food banks and similar charities, they have been touted as a potentially promising avenue for reducing food waste while addressing the challenge of hunger and food insecurity simultaneously^50^. Yet as our results on the demographic make-up of users highlight, food-sharing recipients tend be of lower income but higher education brackets. The high education levels suggests that collectors may have additional resources (e.g., access to technology and social networks) that those most in need of food assistance do not. High education levels have also been documented in participants of other free sharing initiatives (e.g., Timebanks)^39^.

In addition, we find that food typically moves among users who have similar income and education levels. While this might stem from the platform’s location-based character, these results are well aligned with previous work showing that in situations involving free exchanges, people are more likely to engage with strangers of similar status and level of cultural capital^39^. Overall, this suggests that food sharing may not be as effective as has been claimed for addressing food insecurity. However, it should be noted that we used geographic census data to infer users’ income and education levels. Since the average income and education levels in a certain area are not always representative of the individual resident, our findings might be misleading on account of ecological fallacy^51^.

Ultimately, the societal desirability of food sharing will be determined by the magnitude of its social, environmental, and economic implications and by its potential to scale up. A better understanding of “what sells” in a free sharing economy, and the potential impacts of factors such as geographic location, collection restrictions (e.g., time of day), information on the food items’ source (household or retail), and descriptive text and image would likely contribute to the successful expansion of food-sharing practices.

Despite growing interest in the sharing economy, and food sharing in particular^50,52–55^, data-driven analyses of food sharing remain scarce. Here we empirically investigate the full life-cycle environmental impacts of a popular P2P food-sharing platform and examine exchanges from a socio-economic perspective. In addition to measuring food reuse through exchange, we also note implications for both the management of sharing-economy platforms like OLIO as well as the broader universe of sharing platforms.

## Methods

### Dataset construction and analysis

We use a mixed methods approach to empirically investigate food sharing via the popular food-sharing platform OLIO. Our dataset comprised raw data covering all platform activity between April 2017 and October 2018 (inclusive). Although OLIO operates globally, most of the activity is centered in the UK (73%) and Channel Islands (22%). The data provided by OLIO included activity over 19 months for over 30,000 active platform users and was sufficiently rich to allow us to study the types, quantities, and economic value of foods offered and shared, quantify the environmental impacts of food sharing, and examine the flow of food between users from a sociodemographic perspective. A detailed explanation of data processing and preparation for analysis is presented in the SI.

To determine the types of foods offered and shared via the platform we first classified all listings into food categories using a supervised deep learning long short-term memory (LSTM) network. An LSTM is a recurrent neural network that is trained to identify long term dependencies of sequence data. LSTM uses word embedding to map word sequences into numeric vectors, which retain relationships between the words as well as semantic similarities (i.e., similar words or phrases have similar vectors). To create the LSTM network based classifier for OLIO listings, we first manually sorted and tagged over 53,000 OLIO listings into nonfood categories (including supplements such as vitamins and medication, and pet-food) and 13 food categories (see Supplementary Table 6), defined based on properties, such as expected shelf life (e.g., fresh produce and frozen food), storage needs (e.g., dry store), and nutritional value (e.g., snacks, baked goods). We then used this corpus to train, validate, and test the LSTM network (referred to here after as the classifier for clarity) achieving an accuracy level of approximately 0.9, which is typically considered sufficiently high in the field of natural language processing. Finally, all listings (both collected and not collected) were fed into the classifier, which assigned each to one of the predetermined categories. Given our interest in edible food, only the 170,000 unique listings containing food (i.e., categories 1–13 in Supplementary Table 6) and their related users were included in our assessments.

To investigate the viability of food sharing as a strategy for diverting post-retail food waste from disposal and placing it in the hands of interested consumers instead, we examined collection rates in each food category. All listings that had at least one collection (including those offering multiple items) were assumed to be collected in full. To assess the origin of supply and examine the prominence of household food waste verses retail food waste, listings and collection rates were also examined according to the type of user offering them, and whether they were food waste heroes or regular users.

Since most of the food listings do not contain exact weight descriptions, we performed a series of MC simulations (Supplementary Table 7a, b) drawing from an empirical sample of 3300 randomly selected listing for which weights and monetary values were manually assigned based on images and text descriptions.

### Environmental assessment

To examine the full life-cycle environmental impacts of reducing food waste via P2P sharing, we compared the environmental benefits associated with avoided food waste with the environmental burdens associated with added transport required for sharing  (i.e. collectors' travel to and from the pickup sites). While the environmental impacts for both food waste and transport are location specific, in our analysis we focused only on successful exchanges (i.e., collected listings) in the greater London area since this network was by far the most developed, complex, and geographically spaced OLIO network.

Environmental benefits (in metric tonnes of CO_2eq_) were calculated based on the overall mass of food exchanged in the greater London area and a general UK GHG emissions factor for avoided food waste. In line with previous work on food waste recovery^11,56^, we assumed that all foods collected via OLIO fully displaced routine purchases of food in London. Put differently, we presumed that any item collected via OLIO was a perfect substitute for an identical, new food item that would have otherwise been purchased by consumers. See discussion section for more on the implications of this assumption.

### Weights and overall mass of food exchanged

Food waste heroes were more likely to offer multiple items in each listing (e.g., 20 loaves of bread) compared to regular users. Therefore, to preserve the original pattern of their relative contribution we estimated weight-per-listing separately for heroes and regular users (see Supplementary Table 2a, b). Specifically, we split the empirical sample data into groups crossing the 13 food categories (*g*) and the two user types (*u*). Since retailers did not typically provide baby formula or dry tea and coffee, we assumed that listings in these categories always originated from users’ homes, and therefore considered all as if they were offered by regular users. We then ran a series of MC simulations, randomly selecting a group of weights (*w*) from the empirical sample, according to the number of listings exchanged (*n*) via the OLIO platform in each category (*g*) and user type (*u*). In groups where the empirical sample contained fewer than 50 observations, weights (*w*) were randomly drawn from the fitted (i.e., predicted) log-normal distribution of weights instead of the actual empirical sample (see Supplementary Tables 2a, b and 7a). Finally, we summed the mass of food exchanged in all groups and repeated the entire process 10^4^ times to obtain a range of overall mass of food waste avoided (*M*^tot^).

Total avoided mass (*M*^tot^) was calculated using Eq. (1)

1 $$M^{{\mathrm{tot}}} = \mathop {\sum }\limits_{{\mathrm{u}} = 1}^2 \mathop {\sum }\limits_{{\mathrm{g}} = {\mathrm{i}}}^{13} \mathop {\sum }\limits_{{\mathrm{j}} = 1}^{n(g,u)} w,$$

where *M*^tot^, represents the total mass of food exchanged via OLIO in greater London, *u* represents the user group, *g* represents the category group, *n*(*g*, *u*) represents the number of listings exchanged per category and user group, and *w* represents the weight-per listing drawn from the empirical sample or fitted distributions.

### GHG emissions associated with avoided food waste

A literature review revealed a wide range of estimates for food waste related emissions^57,58^. Beyond differences in location, estimates also included several definitions for what constitutes food waste as well as different system boundaries. For example, while some estimates considered only direct impacts from diverting food from disposal^59^, others included embodied emissions, such as those related to food production, processing, transport, and cooling^57,58^, and others considered the COC of abandoning farmland and allowing it to regrow^40^.

Moreover, given that some food items, meat-based products in particular, have substantially higher emission than others, the composition of food waste can also affect emission estimates. Since many of the items offered via the platform contain various food groups (e.g., sandwiches, prepared foods, frozen food, and mixed) it was hard to ascertain the exact composition of items exchanged and build a robust bottom-up emissions factor. For this reason, we applied emissions factors for the average food composition of UK food waste based on values reported by WRAP^56,60^. This UK-specific range of emissions factors (4.0–4.6 t CO_2_eq per ton of food waste) reflects the full life-cycle impacts, including both embodied impacts (i.e., all impacts incurred from farm to fork) as well as direct impacts (i.e., impacts related to end-of-life management of food waste in the UK) of an average kg of food waste in the UK. We adopted a normal distribution with an average value of 4.3 and a coefficient of variation of 10% (*e*_waste_). Although emission factors tend to be location-specific, as a sensitivity analysis we repeated the analysis using a significantly lower emissions factor (2.1 t CO_2_eq per ton of food waste avoided) calculated for the EU as a whole^61^. Results are presented in Supplementary Table 4a, b.

To assess the avoided GHG associated with OLIO exchanges in Greater London, we multiplied the mass of food exchanged in each user-food category group (as derived from Eq. (1)) by a GHG coefficient, randomly selected from the avoided food waste GHG coefficient distribution (*e*_waste_). Repeating this process 10^4^ times for each user-category group and summing all results, we estimated the range of environmental benefits associated with exchanges.

### GHG emissions associated with added travel

Environmental burdens were estimated as a function of the total distance collectors traveled to pick up a food item and the GHG emissions associated with the transport mode. To this end, we constructed six transport scenarios, crossing different travel modes with behavioral assumptions informing the share of trips dedicated solely to food collections (see Table 1). According to recent estimates, only 33% of passenger trips within the London area are done by car while 45% are done by public transport and 21% on foot^45^. Since no information on travel-related activity and modes was available, we conservatively chose to model transport based on the most environmentally intensive transport modes: passenger vehicles and the London bus.

For each collection, road travel distance and travel time (under normal driving conditions) between the collector’s point of origin (assumed to be their default notification location) and the exact collection location (as defined in each listing) were calculated using the STATA (version 14.2) Georoute command^62^. The Georoute command retrieves the road distance, and the time it would take under normal driving conditions to get from point A to point B based on the commercial API HERE (ww.here.com). Much like Google Maps, Waze, or other applications, HERE combines detailed maps with traffic statistics to predict travel routes and times. Since it takes less than 10 min on average to reach a food store in London^63^, exchanges requiring travel longer than 30 min (one-way; *N* = 7567) were excluded leaving 62,570 exchanges for analysis.

To model different travel options, we constructed six travel scenarios crossing transport modes with behavioral assumptions inferring the share of trips dedicated solely to collecting OLIO food (see Table 1). Specifically, scenarios #1–3 assume that all road travel was done by passenger vehicle, while scenarios #4–6 assume all road travel was done via London Bus. Our most behaviorally conservative scenarios (#1, and #4) assume that collectors always made a special, trip back, and forth (i.e., two way) to collect the food item from the provider. Since people often go food shopping “on the way” to other activities, in the next pair of scenarios (#2, and #5) we assumed that 50% of the time, foods were collected on route to another destination (e.g., as part of a daily commute). In our final scenario pair (#3 and #6) we assumed that any travel shorter than 1.6 km (i.e., 1 mile) was done on foot (*N* = 18,072) and thus had no environmental burden in terms of GHG emissions.

For each scenario, we then calculated travel-related burdens by multiplying the overall distance collectors drove, by the full life-cycle GHG emissions associated with the relevant transport mode. Direct emissions coefficients for cars (147 g CO_2_eq per passenger-km) and the local London bus (72 g CO_2_eq per passenger-km) were taken from a recent UK-specific estimate^64^. Since the embodied emissions of cars (i.e., the emissions associated with their production) are ≈20% of direct emissions^65^, we estimated embodied emissions based on the direct emissions and added them to derive the full life-cycle coefficients used to calculate the environmental burdens of travel (*e*_car_ = 184 gCO_2_eq per passenger-km, and *e*_bus_ = 90 g CO_2_eq per passenger-km). As a sensitivity analysis, environmental burdens were also estimated based on higher emission coefficients found in the literature (*e*_car_ = 240, *e*_bus_ = 120^64,66^). Total GHG emissions associated with each scenario travel were estimated using the following equation (Eq. (2))

2 $${\rm{GHG}}^{{\mathrm{scenario}}} = d \times e,$$

where *d* is the overall distance traveled and *e* is the GHG coefficient factor per distance (i.e., *e*_car_ or *e*_bus_).

We used MC simulations (10^4^ times) to derive a distribution for GHG emissions of road travel by varying the relevant GHG coefficient (assuming a normal distribution with a coefficient of variation of 10%). Finally, net environmental benefits were calculated by subtracting the burdens of additional travel from the benefits associated with diverted food waste.

To crudely estimate the minimal displacement rate required to ensure that exchanges of food waste deliver environmental benefits, we divided the GHG burdens associated with added transport by the GHG benefits associated with avoided food production in each of our six scenarios, respectively.

### Carbon opportunity cost

To estimate the COC associated with food sharing, we first calculated the COC associated with 1 kg of food waste in the UK. To this end, we determined the relative contribution each food type made to the overall composition of food waste in the UK (as reported by WRAP^56,60^). In addition, we converted COC factors provided in Searchinger et al.^40^ from production weights (e.g., wheat) to waste weights (e.g., bread, see Supplementary Table 5b). We then multiplied the relative contribution of each food type by its respective COC factor and summed results up to derive the COC per kg of UK food waste. Finally, we multiplied the overall mass of food diverted from disposal via sharing by the UK food waste COC. For more details see Supplementary Table 5 and comments therein.

### Retail value

Retail value was estimated following a similar approach to the one employed to estimate weights. Specifically, using the same empirical sample described previously, we manually assigned each listing with an estimated retail value, based on descriptions, images, and a web search for the retail cost of identical or similar items in the UK. We then used a series of MC simulations to estimate overall retail value following the same procedure we used to estimate food mass.

### Social analysis

Finally, we investigate the social dimensions of OLIO by examining how food moves between UK users living in neighborhoods of varying affluence and education levels (Supplementary Table 8a, b). Since no information on users’ home addresses was available, we used notification locations as defined by each user in their profile as a proxy for providers’ and collectors’ home addresses.

Specifically, we mapped the location of each user in the greater London area (using the STATA package GEOINPOLY^67^, and performed a spatial join with UK official census data to assign each user to a specific Lower Layer Super Output Area (LSOA, by UK 2011 definitions^68^), and the corresponding income and education deciles reported under the Index of multiple deprivation^69^. LSOA is the smallest geographic area defined by the UK office of national statistics, and includes no more than 1000 households by definition. We then created a matrix for the number of exchanges by provider and collector deciles, plotting results into heat maps presented in Fig. 3. Only cases where both users’ locations could be matched to LSOA boundaries were included in the analysis.

### Reporting summary

Further information on research design is available in the Nature Research Reporting Summary linked to this article.

## Supplementary information

Supplementary Information

Reporting Summary

## Data Availability

Aggregated data used in our analysis can be found in the Supplementary Information excel spreadsheet.

## Code availability

The deep learning algorithms using NLP to determine food categories were coded in Matlab (version R2018b). Monte Carlo calculations for food waste amounts and GHG were also conducted in Matlab (version R2018b). Transportation scenarios were calculated using the STATA (version 14.2) Georoute command. The social analysis linking UK census data with food-sharing transactions was performed using the Stata package GEOINPOLY. All relevant code is available from TM and AS upon request.
