# Supplementary material for: Social and environmental analysis of food waste abatement via the peer-to-peer sharing economy
Source: Nat Commun. 2020 Mar 10;11:1156. doi: 10.1038/s41467-020-14899-5 (PMC7064594; doi:10.1038/s41467-020-14899-5)
Supplement: Supplementary file 1 — Supplementary Information [file 41467_2020_14899_MOESM1_ESM.pdf]

Supplementary Information

Social and environmental analysis of food waste abatement via the Peer-to-Peer Sharing Economy

Makov et al.

makovt@bgu.ac.il

Supplementary figures

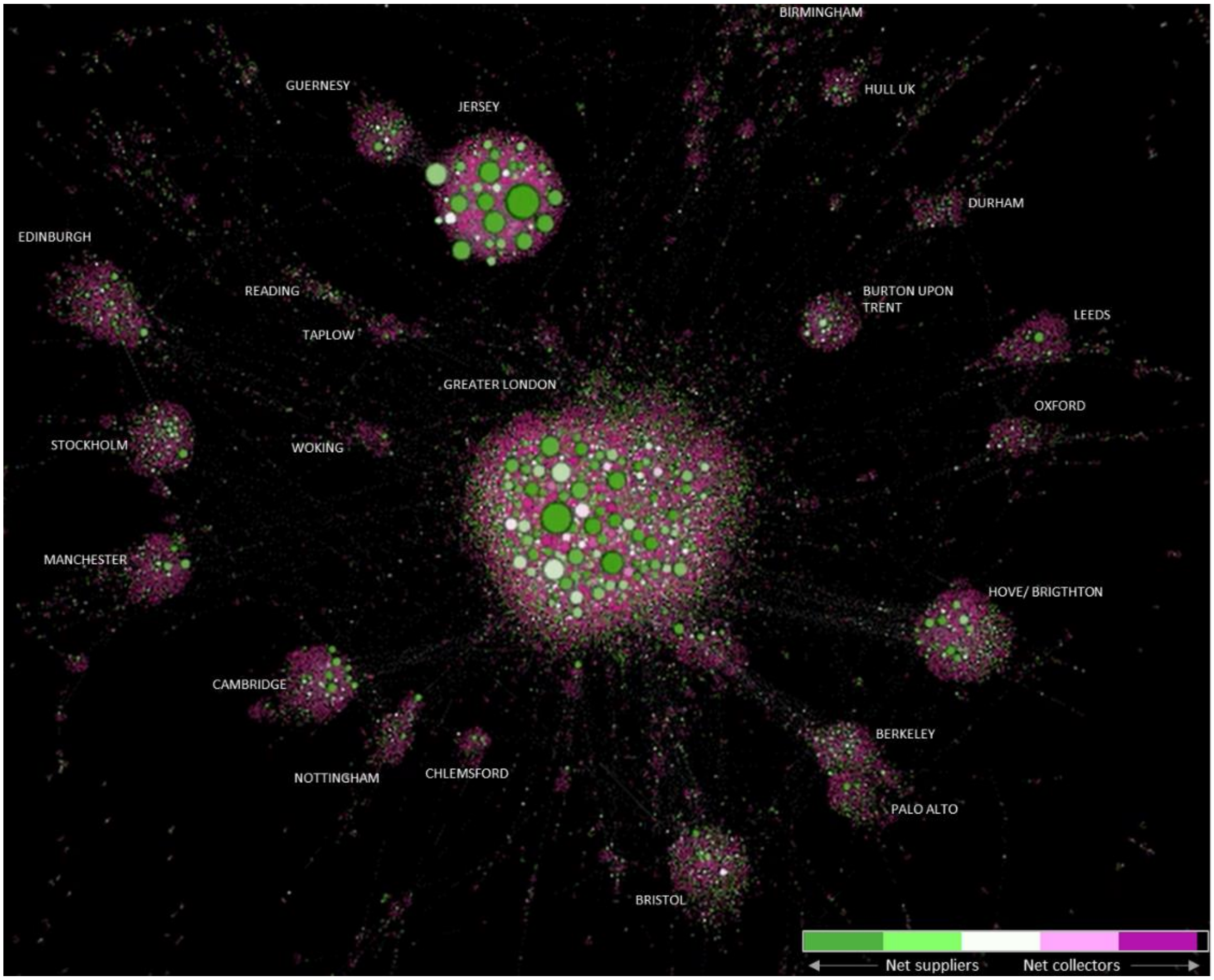

Supplementary Figure 1: Social network of food exchanges. Interconnected social network representing food exchanges via OLIO. Nodes (dots) represent users and lines represent exchanges. Node size is proportional to the number of overall exchanges users engaged. Node color represents the share of items supplied out of overall exchanges from green (net supplier) to purple (net collector).

Supplementary Tables

Makov et al. 2020, Social and environmental analysis of food waste abatement via the Peer-to- Peer Sharing Economy

Table of contents

Table S1a	Avoided food waste and associated GHG emissions, Full OLIO network
Table S1b	Estimated retail value of all foods exchanged, Full OLIO network
Table S2a	Collection rates by category and supplier's Food Waste Hero status (# of listings)
Table S2b	Collection rates by category and supplier's Food Waste Hero status (percent)
Table S3a	Avoided food waste, Greater London area
Table S3b	Added GHG emissions from road travel (tons CO_2eq), Greater London
Table S3c	Avoided vs. added emissions (Greater London)
Table S3d	Number of exchanges (edges) by user group and food category, Greater London area
Table S4a	Sensitivity analysis, Cost benefit (tons of CO_2eq reduced)
Table S4b	Sensitivity analysis, Added GHG emissions from road travel (tons CO_2eq)
Table S4c	Sensitivity analysis, Added GHG emissions from road travel (tons CO_2eq)
Table S5	Carbon Opportunity Cost (COC)
Table S6	Food category definitions and weights based on empirical sample
Table S7a	Monte Carlo results by user type and food category, weights and GHG emissions
Table S7b	Monte Carlo results by user type and food category, economic value
Table S8a	Number of exchanges by UK Income Deciles
Table S8b	Number of exchanges by UK Education Deciles

Table S1a. Avoided food waste and associated GHG emissions, Full OLIO network

	Total mass (tons)	Total GHG associated with avoided food waste (tons CO_2eq)
**5th percentile**	90	386
**Mean**	91	391
**Median**	91	391
**95th percentile**	92	397

Table S1b. Estimated retail value of all foods exchanged, Full OLIO network

	Total retail value (£ 10^6)
**5th percentile**	0.721
**Mean**	0.734
**Median**	0.7
**95th percentile**	0.747

Table S2a. Collection rates by category and supplier's Food Waste Hero status (# of listings)

	Regular users			Food Waste Heroes			Total		
	Not collected	Collected	Total	Not collected	Collected	Total	Not collected	Collected	Total
Baby food	413	149	562	46	35	81	459	184	643
Baked goods	3,463	3,208	6,671	17,781	25,149	42,930	21,244	28357	49,601
Beverages	1,409	1,439	2,848	637	1,097	1,734	2,046	2536	4,582
Dairy	591	494	1,085	627	1,004	1,631	1,218	1498	2,716
Frozen food	260	249	509	69	122	191	329	371	700
Snack & packaged food	1,699	1,634	3,333	965	2,346	3,311	2,664	3980	6,644
Kitchen & pantry staples	9,290	7,481	16,771	4,169	7,753	11,922	13,459	15234	28,693
Mixed	273	323	596	559	1,706	2,265	832	2029	2,861
Prepared food	2,169	1,768	3,937	5,439	12,976	18,415	7,608	14744	22,352
Produce	3,577	3,350	6,927	5,648	14,030	19,678	9,225	17380	26,605
Protein	557	543	1,100	465	1,038	1,503	1,022	1581	2,603
Sandwiches	453	874	1,327	4,565	11,056	15,621	5,018	11930	16,948
Tea & coffee	1,290	1,235	2,525	389	682	1,071	1,679	1917	3,596
**Total**	**25,444**	**22,747**	**48,191**	**41,359**	**78,994**	**120,353**	**66,803**	**101,741**	**168,544**
% of total	*53%*	*47%*		*34%*	*66%*	*71%*	*40%*	*60%*	

Table S2b. Collection rates by category and supplier's Food Waste Hero status (percent)

CATEGORY	Regular Users			Food Waste Heroes			Total		
	Not collected	Collected	% of category total	Not collected	Collected	% of category total	Not collected	Collected	Total
Baby food	73%	27%	87%	57%	43%	13%	71%	29%	0%
Baked goods	52%	48%	13%	41%	59%	87%	43%	57%	29%
Beverages	49%	51%	62%	37%	63%	38%	45%	55%	3%
Dairy	54%	46%	40%	38%	62%	60%	45%	55%	2%
Frozen food	51%	49%	73%	36%	64%	27%	47%	53%	0%
Snack & packaged food	51%	49%	50%	29%	71%	50%	40%	60%	4%
Kitchen & pantry staples	55%	45%	58%	35%	65%	42%	47%	53%	17%
Mixed	46%	54%	21%	25%	75%	79%	29%	71%	2%
Prepared food	55%	45%	18%	30%	70%	82%	34%	66%	13%
Produce	52%	48%	26%	29%	71%	74%	35%	65%	16%
Protein	51%	49%	42%	31%	69%	58%	39%	61%	2%
Sandwiches	34%	66%	8%	29%	71%	92%	30%	70%	10%
Tea & coffee	51%	49%	70%	36%	64%	30%	47%	53%	2%
Total	53%	47%	29%	34%	66%	71%	40%	60%	

Notice that since listings often contain more than one food item, the number of exchanges (i.e. network edges) can be larger than the number of listing collected. The cost benefit analysis is calculated according to the number of exchanges. in the greater London area.

Table S3a. Avoided food waste, Greater London area

	Mean	Median	5th percentile	95th percentile
Total mass (metric tons)	41	41	40	42
Total GHG reduction (metric tons CO_2eq)	176	176	171	181

Table 3b. Added GHG emissions from road travel (tons CO_2eq), Greater London

Travel scenario	Mean	Median	5th percentile	95th percentile	Minimal displacement (mean)
1	89	89	74	103	50%
2	44	44	37	52	25%
3	42	42	35	48	24%
4	43	43	36	51	25%
5	22	22	18	25	12%
6	20	20	17	24	12%

Table S3c. Avoided vs. added emissions Greater London (tons of CO_2eq avoided)

Travel scenario	Mean	Median	5th percentile	95th percentile
1	87	87	72	103
2	132	132	123	140
3	135	134	126	143
4	133	133	124	141
5	154	154	148	160
6	156	156	150	162

Table S3d. Number of exchanges (i.e. edges) by supplier user group and food category, Greater London area

	Regular-user	Food waste hero	Total
baby food	125	22	134
baked goods	1,771	15,127	17,376
beverages	826	924	1,886
dairy	320	773	1,144
frozen food	116	62	193
kitchen store - snack	894	2,136	3,178
kitchen store - store	4,309	5,143	9,804
mixed	193	3,029	3,282
prepared food	1,177	12,694	14,081
produce	1,378	3,072	4,811
protein	741	587	922
sandwiches	1,032	10,995	12,044
tea & coffee	302	517	1,282
**Total**	**13,184**	**55,081**	**70,137**
% of total	*19%*	*79%*	

The sensitivity analysis followed the same steps as the main analysis using different GHG emissions coefficients. For environmental benefits resulting from avoided food waste, we chose a lower value for GHG reductions $e_{waste} =$ 2.1tCO_2eq/ton food waste, as reported by the European Commission (2011) for the whole EU. For environmental costs associated with added road travel, we chose higher emissions factors - e_{car}= 240 gCO_2eq/passenger-km, as reported by European Environment Agency (2018) for the average passenger vehicle in the EU, and e_{bus} =120 gCO_2eq/passenger-km, as reported for a regular bus outside of London by Hill et al. (2018). Since no uncertainty was reported for these coefficients, we used Monte Carlo analysis (10^4) to estimate them (assuming a normal distribution with a coefficient of variation of 10%).

Table 4a. Sensitivity analysis, Cost benefit (tons of CO_2eq reduced)

Travel scenario	Mean	Median	5th percentile	95th percentile
1	-31	-31	-50	-12
2	27	27	17	36
3	30	30	21	40
4	27	27	17	36
5	56	56	51	61
6	57	58	53	63

Table 4b. Sensitivity analysis, Added GHG emissions from road travel (tons CO_2eq)

	Mean	Median	5th percentile	95th percentile
Total mass (tons)	41	41	40	42
Total GHG avoided (tons CO_2eq)	85	85	83	87

Table S4c. Sensitivity analysis, Added GHG emissions from road travel (tons CO_2eq)

Travel scenario	Mean	Median	5th percentile	95th percentile
1	116	116	97	135
2	58	58	48	67
3	54	54	45	63
4	58	58	49	67
5	29	29	24	34
6	27	27	23	31

The calculation of the carbon opportunity cost was preformed by assigning values for COC from Searchinger et al, 2018 to each food group and calculating a weighted average for COC/kg of food waste based on the relative share of each food group in the overall composition of UK food waste (as detailed by WRAP, 2012).

Table 5. Carbon Opportunity Cost

Food type	Mass of edible portion (tonnes/yr)	Mass of inedible portion (tonnes/yr)	Total (tonnes/yr)	Relative share of item out of total food waste mass	Carbon opportunity cost (avg gain and loss) $kgCO_2/kg$ waste weight	Notes on COC	COC per item ($kgCO_2/kg$ waste)
**Fresh vegetable and salads**	**1300000**	**230000**	**1530000**	**0.258**			**0.221**
Potato	710000	0	710000	0.106	1.07	sweet potato tuber	0.113
onion	47000	68000	115000	0.017	0.67	vegetables (all)	0.012
carrot	96000	17000	113000	0.017	0.67	vegetables (all)	0.011
lettuce	57000	11000	68000	0.010	0.67	vegetables (all)	0.007
Other root vegetables	29000	30000	59000	0.009	0.67	vegetables (all)	0.006
cabbage	43000	15000	58000	0.009	0.67	vegetables (all)	0.006
cucumber	43000	7000	50000	0.007	0.67	vegetables (all)	0.005
tomato	46000	3000	49000	0.007	0.67	vegetables (all)	0.005
cauliflower	46000	0	46000	0.007	0.67	vegetables (all)	0.005
broccoli	41000	0	41000	0.006	0.67	vegetables (all)	0.004
pepper	16000	19000	35000	0.005	0.67	vegetables (all)	0.004
mixed vegetables	28000	3000	31000	0.005	0.67	vegetables (all)	0.003
leafy salad	22000	3000	25000	0.004	0.67	vegetables (all)	0.003
mushroom	22000	0	22000	0.003	0.67	vegetables (all)	0.002
leek	10000	11000	21000	0.003	0.67	vegetables (all)	0.002
sweetcorn corn on the cob	7000	9000	16000	0.002	0.67	vegetables (all)	0.002
bean all varieties	8000	4000	12000	0.002	3.87	average of common beans, chickpeas, cowpeas, pigeon peas and lentils	0.007
spring onion	6000	5000	11000	0.002	0.67	vegetables (all)	0.001
all other fresh vegetables and salads	49000	22000	71000	0.011	0.67	vegetables (all)	0.007
	1326000	227000	1553000				
processed potato	77000	1000	78000	0.012	0.67	vegetables (all)	0.008
coleslaw	20000	1000	21000	0.003	0.67	vegetables (all)	0.002
all other processed vegetables and salads	71000	1000	72000	0.011	0.67	vegetables (all)	0.007
	168000	3000	171000				
**Drink**	**700000**	**540000**	**1240000**	**0.182**			**1.127**
tea waste	56000	480000	525000	0.078	12.96	tea leaves (dried)	1.016
carbonated soft drink	230000	0	230000	0.034	0.02	sugar crops	0.001
fruit juice and smoothies	120000	0	120000	0.018	2.77	other fruit temperate	0.050
lager, beer and cider	75000	0	75000	0.011	0.16	wheat grains	0.002
bottled water	54000	0	54000	0.008	0.00		0.000
wine	42000	0	42000	0.006	1.58	other fruit temperate	0.010
squash	39000	0	39000	0.006	0.01	sugar crops	0.000
all other drink	77000	63000	130000	0.019	2.50		0.049
	693000	543000	1215000				
**Fresh fruit**	**300000**	**620000**	**920000**	**0.138**			**0.139**
banana	47000	270000	317000	0.047	1.02	banana	0.048
melon	24000	85000	109000	0.016	1.00	other fruit temperate	0.016
apple	63000	38000	101000	0.015	1.00	other fruit temperate	0.015
orange	29000	69000	98000	0.015	1.00	other fruit temperate	0.015
stone fruit	37000	33000	70000	0.010	1.00	other fruit temperate	0.010
pineapple	10000	59000	69000	0.010	1.00	other fruit temperate	0.010
other citrus	18000	39000	57000	0.009	1.00	other fruit temperate	0.009
soft berry fruit	42000	9000	51000	0.008	1.00	other fruit temperate	0.008
pear	18000	5000	23000	0.003	1.00	other fruit temperate	0.003
all other fresh fruit	13000	13000	26000	0.004	1.00	other fruit temperate	0.004
	301000	620000	921000				
**Meat and fish**	**300000**	**210000**	**510000**	**0.075**			**6.826**
poultry chicken turkey duck	100000	150000	250000	0.037	19.61	poultry meat	0.732
pork ham bacon	100000	22000	122000	0.018	25.54	pork meat	0.465
beef	47000	4000	51000	0.008	371.06	beef meat	2.827
fish and shellfish	23000	9000	32000	0.005			

lamb	7000	7000	14000	0.002	664.17	sheep/goat meat	1.389
all other meat and fish	23000	12000	35000	0.005	270.09	all meat	1.412
	300000	204000	504000				
**Bakery**	**500000**	**1000**	**501000**	**0.075**			**0.104**
standard bread	410000	0	410000	0.061	1.33	wheat grain	0.082
specialty bread	43000	0	43000	0.006	2.15	average of wheat, barely, and sorghum grains	0.014
morning goods	18000	0	18000	0.003	0.80	wheat grain	0.002
all other bakery	29000	0	29000	0.004	1.43		0.006
	500000	0	500000				
**Dairy and eggs**	**410000**	**59000**	**469000**	**0.070**			**0.809**
milk	290000	0	290000	0.043	6.64	cow milk	0.288
eggs	21000	59000	80000	0.012	11.05	eggs	0.132
yoghurt	51000	0	51000	0.008	6.64		0.051
cheese	32000	0	32000	0.005	66.40		0.317
cream and crème fraiche	16000	0	16000	0.002	6.64	as yoghurt	0.016
all other dairy and eggs	2000	0	2000	0.000	19.47		0.006
	412000	59000	471000				
**meals (home made and pre=prepared)**	**420000**	**1000**	**421000**	**0.062**			**5.135**
composite meal	260000	0	260000	0.039	107.46	mixed ingredients	4.174
soup	70000	0	70000	0.010	0.34		0.004
sandwich	51000	0	51000	0.008	51.95		0.396
savoury products	35000	0	35000	0.005	107.46	assuming a mixed composition of grains and dairy and meat	0.562
	416000	0	416000				
**All other food and drink**	**780000**	**360000**	**1140000**	**0.141**			**4.947**
cakes gateau doughnuts pastries	75000	0	75000	0.011	4.48	mixed ingredients of sugar, wheat and eggs	0.050
all other cake and desserts	69000	0	69000	0.010	4.48	mixed ingredients of sugar, wheat and eggs	0.046
	144000	0	144000				
breakfast cereal	60000	0	60000	0.009	1.12	several cereals and sugars	0.010
rice	41000	0	41000	0.006	0.96	rice grains (rough)	0.006
pasta	31000	0	31000	0.005	2.00	wheat grains	0.009
all other staple foods	10000	0	10000	0.001	1.36		0.002
	142000	0	142000				
cook in sauce	41000	0	41000	0.006			
gravy	9000		9000	0.001			
all other condiments sauces herbs and spices	83000	2000	85000	0.013			
	133000	2000	135000				
fat	11000	0	11000	0.002			
all other oil	59000	3000	62000	0.009	8.79	average of soybean, palm, palm kernel, canola, sunflower, groundnut, and maize oil	0.081
	70000	3000	73000				
savoury snacks	21000	1000	22000	0.003	107.46	mixed ingredients as composite meal	0.353
sweet biscuits	18000	0	18000	0.003	1.31	mixed ingredients of flour, sugar and oil	0.004
chocolate and sweets	18000	0	18000	0.003	6.68	mixed ingredients including sugar, milk and cocoa	0.018
all other confectionary and snacks	2000	0	2000	0.000	4.00		0.001
	59000	1000	60000				
mixed semi solid food	1000	250000	251000	0.037	107.46	assuming mixed ingredients of composite meals	4.029
drainings from canned food	20000	97000	117000	0.017	0.00	0	0.000
remaining other	21000	0	21000	0.003	107.46	assuming mixed ingredients of composite meals	0.337
	42000	347000	389000				
	590000	353000	943000				
**Total per group**	**4710000**	**2021000**	**6731000**	1	g food waste) by per group calculation		**19.308**
Total per item	4706000	2009000	6694000	1	kg food waste) by per item calculation		**19.308**
				contribution from total of excluded items with unknown COC 0.027			

Table S5b. from waste weight to production

Item	Conversion	Source
bread to wheat	0.60	A. Korsaeth etal. (2012) Environmental life cycle assessment of cereal and bread production in Norway, Acta Agriculturae Scandinavica, Section A – Anima
pasta to grains	0.90	https://bakerpedia.com/processes/moisture/
rice to dried rice	0.40	https://www.sciencedirect.com/science/article/pii/S0271531705804725
legumes ready-to-eat to dried legumes	0.35	https://link.springer.com/article/10.1007/BF01092032
fruit juice average pressing yield	2.77	http://randd.defra.gov.uk/Document.aspx?Document=11123_20130430Annexes1,2,5,6FINALMarch2013.pdf
wine to grapes	1.58	https://grapesandwine.cals.cornell.edu/newsletters/appellation-cornell/2011-newsletters/issue-8/conversion-factors-vineyard-bottle/
beef	2.40	https://extension.tennessee.edu/publications/Documents/PB1822.pdf
pork	1.74	https://www.oda.state.ok.us/food/fs-hogweight.pdf
poultry	1.77	https://pdfs.semanticscholar.org/63e8/c0a185ddbde1ed626e8f73c54128730df43f.pdf
goat	3.33	http://agriculture.vic.gov.au/agriculture/livestock/goats/production/meat-and-offal-yields-of-goats
conversion from beer to wheat	0.07	https://link.springer.com/article/10.1007/s11367-016-1028-6

Table S6. Food category definitions and weights based on empirical samples

Category	Description	[1] Weight by group (g) based on data samples	
		Regular users	Food waste heroes
Baby food	Liquid baby formula, baby formula powder	N=16<br>ln(w) ~N (6.1, 0.7)	[2] NA
Baked Goods	Breads, pastries, cakes, cookies, flatbreads, etc. from bakeries and markets. Includes both savory and sweet baked goods. Can include mixed postings if all (or a vast majority of) items also fall within this category.	N=301<br>Mean = 972<br>Median =400<br>SD = 3838	N= 399<br>Mean = 572<br>Median =400<br>SD = 763
Beverages	Liquid beverages, Milk and non-dairy variants thereof (e.g. soy milk).	N= 160<br>Mean = 2357<br>Median =1000<br>SD = 4544	N=61<br>Mean = 1572<br>Median =1000<br>SD = 2791
Dairy	Butter, yogurt, ice cream, cheese, and other dairy products	N=58<br>Mean = 7.3<br>Median =500<br>SD = 857	N= 34<br>ln(w) ~ N (6.3, 0.9)
Frozen Food	Mainly frozen meals and other ready-to-cook items. Also includes other food items that must be kept frozen but that do not fall into any other category.	N= 42<br>ln(w) ~N (6.2, 1.0)	N=19<br>ln(w) ~N (6.9, 0.9)
Snacks & packaged	Crackers, chips (crisps), popcorn, and other snack foods.	N= 124<br>Mean = 282<br>Median =200<br>SD = 302	N= 63<br>Mean = 761<br>Median =250<br>SD = 3008
Kitchen & pantry staples	All pantry items, incl. baking ingredients, canned foods, bottles, oils, condiments, spices, grains, and open (i.e. perishable) examples thereof.	N=683<br>Mean = 722<br>Median =335<br>SD = 1856	N=181<br>Mean = 478<br>Median =350<br>SD = 570
Mixed	Posts that include substantial amounts from more than one of the food categories listed above	N=30<br>ln(w) ~N (7.6, 1.4)	N=33<br>ln(w) ~N (7.5, 1.5)
Prepared Food	Perishable food that has been prepared for consumption. Includes cooked grains, meals, and chopped vegetables. Excludes sandwiches and baked goods.	N= 113<br>Mean = 587<br>Median =320<br>SD = 906	N=204<br>Mean = 693<br>Median =400<br>SD = 1411
Produce	Fresh fruits, vegetables, and herbs.	N= 332<br>Mean = 844<br>Median =450<br>SD = 1782	N=141<br>Mean = 1661<br>Median =500<br>SD = 3084
Protein	Meat, eggs, and meat substitutes (e.g. tofu)	N= 75<br>Mean = 795<br>Median =440<br>SD = 1110	N=41<br>ln(w) ~N (6.3, 1.1)
Sandwiches	Sandwiches, wraps, ciabatta, and other like items.	N=64<br>Mean = 643<br>Median =395<br>SD = 639	N=142<br>Mean = 608<br>Median =350<br>SD = 980
Tea & Coffee	Dry tea & coffee, coffee pods	N=103<br>Mean = 243<br>Median =120<br>SD = 434	NA

[1] In groups where the empirical samples are N<50 we report statistics of the fitted lognormal distributions from which weights were drawn.
For groups where the empirical samples are N>50 we report mean, mode, and SD of empirical distribution from which weights were drawn (in grams).
[2] This group of samples by food waste heroes had only a few items. Therefore, we assumed its distribution is identical to that of regular users.

Table S7a. Monte Carlo results by user type and food category, weights and GHG emissions

		Baby food	Baked goods	Beverages	Dairy	Frozen food	Snack & packaged food	Kitchen & pantry staples	Mixed	Prepared food	Produce	Sandwiches	Tea & coffee	Protein
**Food waste heroes**	available observations (N)	0	339	61	34	19	63	181	33	204	141	142	0	41
Weight per listing (g)	Mean	0	579	1631	900	1504	729	480	5592	657	1591	604	0	1021
	Median	0	400	1000	571	977	250	350	1843	390	500	350	0	535
	5th percentile	0	100	100	124	211	90	80	144	110	100	195	0	81
	95th percentile	0	2000	5000	2735	4486	1320	2000	21668	1800	6900	1500	0	3379
Mass (tons)	Mean	0.00	14.55	1.79	0.90	0.18	1.71	3.72	9.54	8.52	22.32	6.67	0.00	1.06
	Median	0.00	14.55	1.78	0.90	0.18	1.70	3.72	9.51	8.52	22.31	6.67	0.00	1.06
	5th percentile	0.00	14.35	1.63	0.85	0.15	1.49	3.64	8.63	8.29	21.76	6.50	0.00	0.98
	95th percentile	0.00	14.76	1.95	0.96	0.22	1.94	3.81	10.50	8.76	22.89	6.85	0.00	1.15
Total GHG avoided (tons)	Mean	0.00	62.58	7.69	3.89	0.79	7.35	16.01	41.01	36.65	95.97	28.70	0.00	4.56
	Median	0.00	62.57	7.67	3.89	0.78	7.32	16.00	40.91	36.64	95.96	28.69	0.00	4.55
	5th percentile	0.00	61.72	7.01	3.65	0.66	6.40	15.65	37.08	35.67	93.53	27.96	0.00	4.19
	95th percentile	0.00	63.45	8.40	4.14	0.94	8.35	16.37	45.19	37.67	98.41	29.46	0.00	4.94
**Regular users**	available observations (N)	16	301	160	58	42	124	683	30	113	332	64	103	75
Weight per listing (g)	Mean	566	966	2311	699	751	283	746	4992	581	839	642	241	799
	Median	455	400	1000	500	481	200	330	1953	320	450	400	120	440
	5th percentile	151	125	170	125	98	50	30	201	99	50	195	16	100
	95th percentile	1346	2400	9000	2100	2256	800	2157	18473	1830	2500	1902	720	4032
Mass (tons)	Mean	0.10	3.10	3.33	0.35	0.19	0.46	5.58	1.61	1.03	2.81	0.56	0.46	0.43
	Median	0.10	3.09	3.32	0.35	0.19	0.46	5.58	1.59	1.03	2.81	0.56	0.46	0.43
	5th percentile	0.10	2.77	3.05	0.32	0.17	0.44	5.31	1.30	0.97	2.64	0.53	0.43	0.39
	95th percentile	0.11	3.47	3.61	0.38	0.21	0.48	5.86	2.01	1.09	2.99	0.59	0.49	0.48
Total GHG avoided (tons)	Mean	0.45	13.34	14.30	1.49	0.80	1.99	24.00	6.93	4.42	12.09	2.41	1.99	1.87
	Median	0.45	13.29	14.30	1.48	0.80	1.99	23.99	6.82	4.41	12.07	2.41	1.99	1.86
	5th percentile	0.41	11.88	13.13	1.36	0.71	1.91	22.82	5.61	4.16	11.36	2.28	1.86	1.68
	95th percentile	0.49	14.93	15.53	1.63	0.91	2.08	25.19	8.67	4.67	12.86	2.55	2.12	2.05

Table S7b. Monte Carlo results by user type and food category, economic value

		Baby food	Baked goods	Beverages	Dairy	Frozen food	Snack & packaged food	Kitchen & pantry staples	Mixed	Prepared food	Produce	Sandwiches	Tea & coffee	Protein
**Food waste heroes**	available observations (N)	0	339	61	34	19	63	181	33	204	141	142	0	41
Value per listing (£)	Mean	0.00	6.41	6.75	9.70	7.45	5.10	5.98	66.95	7.41	5.54	8.05	0.00	7.70
	Median	0.00	3.00	2.00	5.26	5.19	2.00	3.00	22.59	5.00	2.20	6.00	0.00	4.35
	5th percentile	0.00	0.80	0.55	0.83	1.29	0.70	0.60	1.94	1.10	0.81	2.10	0.00	0.71
	95th percentile	0.00	18.00	20.00	32.91	20.91	18.00	15.00	262.66	24.00	26.00	20.00	0.00	25.66
Total value (10^6 £)	Mean	0.000	0.161	0.007	0.010	0.001	0.012	0.046	0.114	0.096	0.078	0.089	0.000	0.008
	Median	0.000	0.161	0.007	0.010	0.001	0.012	0.046	0.114	0.096	0.078	0.089	0.000	0.008
	5th percentile	0.000	0.157	0.006	0.009	0.001	0.011	0.043	0.104	0.095	0.076	0.088	0.000	0.007
	95th percentile	0.000	0.166	0.008	0.011	0.001	0.013	0.050	0.125	0.097	0.079	0.090	0.000	0.009

		Baby food	Baked goods	Beverages	Dairy	Frozen food	Snack & packaged food	Kitchen & pantry staples	Mixed	Prepared food	Produce	Sandwiches	Tea & coffee	Protein
**Regular users**	available observations (N)	16	301	160	58	42	124	683	30	113	332	64	112	75
Value per listing (pounds UK)	Mean	5.02	5.21	6.17	4.26	5.41	3.06	3.88	24.15	4.89	2.02	9.26	6.73	5.15
	Median	4.11	2.00	3.00	2.00	3.47	2.00	2.00	13.07	2.80	1.47	5.00	3.98	3.40
	5th percentile	1.40	0.70	0.60	1.00	0.71	0.65	0.40	2.08	0.80	0.34	2.35	0.84	0.85
	95th percentile	11.80	20.00	25.00	9.50	16.89	10.00	12.00	79.99	20.00	6.00	27.00	14.00	15.60
Total value (10^6 pounds UK)	Mean	0.001	0.017	0.009	0.002	0.001	0.005	0.029	0.008	0.009	0.007	0.008	0.013	0.003
	Median	0.001	0.017	0.009	0.002	0.001	0.005	0.029	0.008	0.009	0.007	0.008	0.013	0.003
	5th percentile	0.001	0.016	0.008	0.002	0.001	0.005	0.028	0.007	0.008	0.007	0.008	0.012	0.003
	95th percentile	0.001	0.018	0.010	0.002	0.002	0.005	0.031	0.009	0.009	0.007	0.009	0.014	0.003

Table S8a. UK exchanges by Income Decile (where 1 is most deprived 10% of LSOAs)

		collectors										
		1	2	3	4	5	6	7	8	9	10	Total
suppliers	1	2,178	2,506	1,542	1,146	517	543	340	386	219	488	9,865
	2	3,047	4,858	3,580	1,954	2,001	1,223	941	878	348	801	19,631
	3	1,286	2,515	3,540	1,716	1,774	1,003	620	714	388	631	14,187
	4	1,516	2,424	1,923	2,852	1,128	898	812	577	452	785	13,367
	5	581	878	951	594	1,303	645	547	575	254	697	7,025
	6	548	991	941	802	751	954	483	388	320	400	6,578
	7	462	764	1,202	773	836	1,009	1,259	765	490	837	8,397
	8	271	396	657	389	609	435	366	493	215	318	4,149
	9	207	306	468	263	659	507	385	335	592	556	4,278
	10	348	537	1,041	650	672	640	814	496	393	2,255	7,846
	Total	10,444	16,175	15,845	11,139	10,250	7,857	6,567	5,607	3,671	7,768	95,323

Table S8b. UK exchanges by Education Decile (where 1 is most deprived 10% of LSOAs)

		collectors										
		1	2	3	4	5	6	7	8	9	10	Total
suppliers	1	922	452	385	416	154	137	137	329	120	151	3,203
	2	472	987	370	365	210	401	197	259	145	211	3,617
	3	207	427	1,187	873	963	866	828	610	539	641	7,141
	4	165	324	829	1,718	1,383	1310	871	885	573	1078	9,136
	5	180	595	695	880	1,917	1689	1034	1000	774	907	9,671
	6	292	764	1042	1663	1851	3709	1974	1856	1422	1709	16,282
	7	163	419	841	828	1315	1,377	1,412	1226	827	1234	9,642
	8	120	424	818	978	1652	1681	1712	2049	1752	2611	13,797
	9	151	318	612	709	1118	1215	920	1387	1374	1837	9,641
	10	154	366	455	777	1081	1683	1120	1438	1807	4,312	13,193
	Total	2,826	5,076	7,234	9,207	11,644	14,068	10,205	11,039	9,333	14,691	95,323

Supplementary notes

Supplementary Note 1. Introduction

OLIO is a UK based food sharing startup founded in 2015 by Tessa Clarke and Sasha Celestial One. The platform can be accessed via Web browsers (https://olioex.com/) or dedicated smartphone apps and is freely available through the Apple and Samsung application stores. At the time of writing the platform had over 700,000 registered users worldwide. While the main focus is on food, the platform also has sections for sharing furniture, clothes etc. In addition to regular users, offering whatever food waste they have in their houses, OLIO also operates a network of individual volunteers, called 'food waste heroes', who collect food surplus from local businesses such as delis and bakeries, and offers them for collection via the network. OLIO's business model is to charge local businesses for certifying that they are 'zero-food waste' operators. Critically, while the startup is a for-profit enterprise, users are free to post or collect as many items as they wish, and all exchanges facilitated via the platform are currently free of charge.

Supplementary Note 2. Classification into food categories

We developed the classification scheme using an iterative and inductive process. Each of the 53,463 OLIO postings that comprised the training set was first manually categorized by one of four researchers. These categories were not predetermined; the researchers were free to create categories as needed. Efforts were made to select food categories that appeared to be mutually exclusive and sufficiently encompassing, which required a degree of iteration even within the first pass. The nature of the data posed some challenges for effective categorization. For one, information about each posting was exclusively in open text fields, which means there was no standard format in terms of text structure or content. Furthermore, the international scope of OLIO's user base means that there were many food products and slang terms used that were unfamiliar to the researchers.

Nevertheless, this initial pass through the data set resulted in 448 unique tags, which were then harmonized and condensed to 19 categories by a single researcher. After identifying systemic errors and inconsistencies, some of which stemmed from the open field format of the listings and others which stemmed from differences of the three initial coders, a second pass by a single researcher adjusted and reduced the categories down to 15. This scheme was used to train the classifier and test accuracy of the algorithm. Based on results from this test, a final scheme of 13 food categories (see Supplementary Table 6 in the SI excel) and 3 non-food categories (supplements, pet foods and NA) was imposed on the training set. This is the classification used in this paper

Supplementary Note 3. Sensitivity analysis- Environmental cost benefit

The sensitivity analysis followed the same steps as the main analysis differing only in the GHG emissions coefficients used for calculations. For environmental benefits resulting from avoided food waste, we chose a lower value for GHG reductions, $e_{waste} =$ 2.1tCO_2eq/ton for food waste, as reported by the European Commission (2011) for the whole EU. For environmental costs associated with added road travel, we chose higher emissions factors - e_{car}= 240

gCO_2eq/passenger-km, as reported by European Environment Agency (2018) for the average passenger vehicle in the EU, and e_{bus} =120 gCO_2eq/passenger-km, as reported for a regular bus outside of London by Hill et al. (2018). Since no uncertainty was reported for these coefficients, we used Monte Carlo analysis (10^4) to estimate them (assuming a normal distribution with a coefficient of variation of 10%). Result for sensitivity analysis are presented in the supplementary information (see Supplementary Tables 4a-c in the SI excel).
