# Supplementary material for: Social and environmental analysis of food waste abatement via the peer-to-peer sharing economy
Source: Nat Commun. 2020 Mar 10;11:1156. doi: 10.1038/s41467-020-14899-5 (PMC7064594; doi:10.1038/s41467-020-14899-5)
Supplement: Supplementary file 2 — Reporting Summary [file 41467_2020_14899_MOESM2_ESM.pdf]

nature research

Corresponding author(s): Tamar Makov

Last updated by author(s): 18-01-2020

Reporting Summary

Nature Research wishes to improve the reproducibility of the work that we publish. This form provides structure for consistency and transparency in reporting. For further information on Nature Research policies, see Authors & Referees and the Editorial Policy Checklist .

Statistics

For all statistical analyses, confirm that the following items are present in the figure legend, table legend, main text, or Methods section.

n/a	Confirmed	
x		The exact sample size (n) for each experimental group/condition, given as a discrete number and unit of measurement
x		A statement on whether measurements were taken from distinct samples or whether the same sample was measured repeatedly
x		The statistical test(s) used AND whether they are one- or two-sided<br>*Only common tests should be described solely by name; describe more complex techniques in the Methods section.*
x		A description of all covariates tested
	x	A description of any assumptions or corrections, such as tests of normality and adjustment for multiple comparisons
	x	A full description of the statistical parameters including central tendency (e.g. means) or other basic estimates (e.g. regression coefficient) AND variation (e.g. standard deviation) or associated estimates of uncertainty (e.g. confidence intervals)
x		For null hypothesis testing, the test statistic (e.g. F, t, r) with confidence intervals, effect sizes, degrees of freedom and P value noted<br>*Give P values as exact values whenever suitable.*
x		For Bayesian analysis, information on the choice of priors and Markov chain Monte Carlo settings
x		For hierarchical and complex designs, identification of the appropriate level for tests and full reporting of outcomes
x		Estimates of effect sizes (e.g. Cohen's d, Pearson's r), indicating how they were calculated

Our web collection on statistics for biologists contains articles on many of the points above.

Software and code

Policy information about availability of computer code

Data collection	No software was used in collecting the data. The data was provided by OLIO via SQL.
Data analysis	The deep learning algorithms using NLP to determine food categories were coded in Matlab (version R2018b). Monte Carlo calculations for food waste amounts and GHG were also conducted in Matlab (version R2018b). Transportation scenarios were calculated using the STATA (version 14.2) Georoute command. The social analysis linking UK census data with food sharing transactions was preformed using the STATA package GEOINPOLY. The network analysis was performed using Gephi and STATA. All relevant code is available from TM and AS upon request

For manuscripts utilizing custom algorithms or software that are central to the research but not yet described in published literature, software must be made available to editors/reviewers. We strongly encourage code deposition in a community repository (e.g. GitHub). See the Nature Research guidelines for submitting code & software for further information.

Data

Policy information about availability of data

All manuscripts must include a data availability statement. This statement should provide the following information, where applicable:

- Accession codes, unique identifiers, or web links for publicly available datasets
- A list of figures that have associated raw data
- A description of any restrictions on data availability

The raw data detailing food sharing transactions can not be made publicly available since it holds users' personal information and is a proprietary of OLIO. Aggregated data used in our analysis can be found in the Supplementary Information excel spreadsheet.

Field-specific reporting

Please select the one below that is the best fit for your research. If you are not sure, read the appropriate sections before making your selection.

☐ Life sciences ☒ Behavioural & social sciences ☐ Ecological, evolutionary & environmental sciences

For a reference copy of the document with all sections, see nature.com/documents/nr-reporting-summary-flat.pdf

Behavioural & social sciences study design

All studies must disclose on these points even when the disclosure is negative.

Study description	This study is a quantitative analysis using various different mathematical tools and techniques which 1) categorize the types of food shared (deep learning and natural language processing); 2) estimates the mass of foods shared (Monte Carlo); 3) estimates GHG emissions (or avoided emissions) associated with food exchanges (Monte Carlo, GEOROTUE calculations); and 4) infers on the socio economic strata of the users involved in this network of food sharing.
Research sample	The dataset upon which this analysis was conducted was provided by OLIO detailing the listings and transactions of food sharing across the platform.
Sampling strategy	The entire raw dataset provided by OLIO for a time period ranging between 04/2017-10/2018 was used
Data collection	The raw data was provided by OLIO.
Timing	The dataset includes activity of food sharing from April 2017 till Oct 2018.
Data exclusions	Listings that did not contain food items or were unidentified (e.g. gibbrish) were excluded.
Non-participation	N/A
Randomization	Because the the actual amount of food items exchanged across the network were unavailable we used Monte Carlo randomization to try and infer on those quantities (see Manuscript for more details).

Reporting for specific materials, systems and methods

We require information from authors about some types of materials, experimental systems and methods used in many studies. Here, indicate whether each material, system or method listed is relevant to your study. If you are not sure if a list item applies to your research, read the appropriate section before selecting a response.

Materials & experimental systems

n/a	Involved in the study	
☒	☐	Antibodies
☒	☐	Eukaryotic cell lines
☒	☐	Palaeontology
☒	☐	Animals and other organisms
☒	☐	Human research participants
☒	☐	Clinical data

Methods

n/a	Involved in the study	
☒	☐	ChIP-seq
☒	☐	Flow cytometry
☒	☐	MRI-based neuroimaging
